# Oncolytic adenovirus MEM-288 encoding membrane-stable CD40L and IFNβ induces an anti-tumor immune response in high grade serous ovarian cancer

**DOI:** 10.1016/j.neo.2024.101056

**Published:** 2024-09-13

**Authors:** Pamela N. Peters, Regina S. Whitaker, Felicia Lim, Shonagh Russell, Elizabeth A. Bloom, Justin Pollara, Kyle C. Strickland, Mark J. Cantwell, Amer Beg, Andrew Berchuck, Scott Antonia, Rebecca A. Previs

**Affiliations:** a Duke University Department of Obstetrics and Gynecology; b Duke University Department of Pharmacology and Cancer Biology; c Duke University Department of Surgery; d Duke University School of Medicine; e Duke University Department of Pathology; f Memgen, Inc; g Moffitt Cancer Center Department of Immunology; h Labcorp Oncology, Durham, NC, USA

**Keywords:** Immunotherapy, Ovarian cancer, Oncolytic virus, Ascites, ELISPOT, ELISA, Intraperitoneal, Microenvironment

## Abstract

Single agent immune checkpoint inhibitors have been ineffective for patients with advanced stage and recurrent high grade serous ovarian cancer (HGSOC). Using pre-clinical models of HGSOC, we evaluated the anti-tumor and immune stimulatory effects of an oncolytic adenovirus, MEM-288. This conditionally replicative virus encodes a modified membrane stable CD40L and IFNβ. We demonstrated this virus successfully infects HGSOC cell lines and primary human ascites samples *in vitro*. We evaluated the anti-tumor and immunostimulatory activity *in vivo* in immune competent mouse models. Intraperitoneal delivery of MEM-288 decreased ascites and solid tumor burden compared to controls, and treatment generated a systemic anti-tumor immune response. The tumor microenvironment had a higher proportion of anti-tumor macrophages and decreased markers of angiogenesis. MEM-288 is a promising immunotherapy agent in HGSOC, with further pre-clinical studies required to understand the mechanism of action in the peritoneal microenvironment and clinical activity in combination with other therapies.

## Introduction

Advances in therapeutics for epithelial ovarian cancer through surgery, chemotherapy, and the incorporation targeted therapies have resulted in over half of patients surviving more than five years [1]. However, while surgery and chemotherapy for most patients achieves remission, defined by no evidence of cancer by imaging or lab work, almost all patients develop recurrence and resistance to therapy. Intra-abdominal dissemination of cancer and accumulation malignant ascites ultimately affects quality of life and leads mortality through malnutrition and bowel obstruction. Intraperitoneal treatment have been historically used in ovarian cancer and continue to be developed due to this pattern of spread [[2], [3], [4]].

Immunotherapy introduced a new paradigm for the treatment of patients with chemo-resistant cancers. Immune checkpoint inhibitors have meaningfully impacted outcomes in multiple tumor types, particularly lung cancer [5] and melanoma [6,7]. In gynecologic malignancies, immunotherapy is now part of standard frontline treatment for advanced cervical and endometrial cancer [[8], [9], [10], [11], [12]]. However, there has been limited success of immune checkpoint blockade in epithelial ovarian cancer [[13], [14], [15]], despite the evidence that ovarian cancer is an immunogenic cancer [[16], [17], [18]]. This dichotomy has been ascribed to the low diversity of tumor neo-antigens, few coding mutations, and dysregulation of T-cell trafficking to the immune microenvironment [19]. Novel strategies are required to effectively direct the immune system against epithelial ovarian cancer.

Viral immunotherapy, including oncolytic viruses, has demonstrated activity in immunogenic tumors [20]. Oncolytic viruses selectively replicate in cancer cells leaning to cell lysis without cytotoxicity to normal cells and prime the immune system through release of tumor antigens and antiviral immune responses against infected cells. Oncolytic viruses are also routine armed through insertion of immunostimulatory transgenes to further potentiate antitumor activity [21,22].

MEM-288 is a novel oncolytic virus engineered with well-characterized viral genome deletions in E1A, E1B and E3 regions that impart conditional tumor replication [23]. MEM-288 encodes two potent immune agonists: IFNβ and a recombinant membrane-stable chimeric form of CD40L (MEM40) [24]. This agonist combination has been demonstrated to be better than either agonist individually to activate conventional dendritic cells type 1 (cDC1) crucial for CD8+ T-cell cross-priming, and to increase tumor-antigen reactive CD8+ T-cells [[25], [26], [27]]. MEM-288 is currently being studied in a phase 1 first-in-human trial as a standalone agent and in combination with immune checkpoint inhibitors for patients with non-small cell lung (NSCLC) that have progressed with standard chemotherapy and immune checkpoint inhibitor therapy. Results have showed that intratumoral injection of MEM-288 leads to tumor shrinkage and with systemic anti-tumor T-cell immunity [28].

Given MEM-288′s ability to strongly stimulate systemic anti-tumor responses, we aimed to evaluate the ability of MEM-288 to infect epithelial ovarian cancer cells, and assess the effects of a novel intraperitoneal route of administration on the local immune microenvironment, systemic immune response, and anti-tumor activity.

## Materials and methods

### Cell cultures

We obtained human ovarian cancer cell lines (Jhos2, TykNu) from the Duke Gynecology/Oncology Bank (Durham, NC). Jhos2 cells were cultured in DMEM/F12 media with 10 % fetal bovine serum (FBS). TykNu cells were cultured in RPMI media with 10 % FBS. STOSE and STOSE-luc cells were a gift from Dr. Barbara Vanderhyden (University of Ottawa) [40]. IG10 and IG10-luc cells were a gift from Dr. Guillermo Armaiz (Ponce Health Sciences University, Ponce, PR) [41]. STOSE and IG10 cells were cultured in DMEM media supplemented with 4 % FBS. All cell lines were maintained at 37 °C in a humidified incubator at 5 % CO2. Experiments were performed at 40-60 % confluence.

### Human ascites

Ascites samples from patients with HGSOC were obtained after informed consent. The ascites fluid was separated from the ascites cells after centrifugation and frozen in 90 % fetal calf serum and 10 % DMSO cryopreservation solution at −150 °C until used. After thawing, human ascites cells were cultured in RPMI media supplemented with 10 % FBS.

### Adenovirus

MEM-288 and Adv-GFP virus were obtained as a gift from Dr. Amer Beg (Moffitt Cancer Center) and Memgen[29]. MEM-288 was manufactured and stored at -80C a concentration of 0.62 × 10^10^ PFU/mL (batch 1) and 1.2 × 10^11^ PFU/mL (batch 2). Adv-GFP was manufactured and stored in the same conditions at a concentration of 3.3 × 10^10^ PFU/mL (batch 1) and 1.5 × 10^11^ (batch 2).

### IFNβ ELISA

To assess IFNβ secretion, cells [TykNu 7.5 × 10^3^; Jhos2 1 × 10^4^; STOSE-luc 5 × 10^3^, IG10-luc 5 × 10^3^; ascites 1 × 10^4^] were plated in triplicate in each well of a 96 well plate and maintained overnight. The IC_50_ MOI at 48 hours as determined by cell viability assay was selected for each cell line (TykNu 37; Jhos2 173; STOSE-luc 89; IG10-luc 64; ascites 74). MEM-288, Adv-GFP, or PBS was added to culture media. After 24 hours, media was aspirated and cells washed with PBS. Culture media was replaced, and the cells were incubated again for 24 hours at 37⁰C. IFNβ levels in the supernatant were assessed with a human IFNβ specific ELISA (PBL Assay Science, #41410). Experiments were performed in biologic triplicates for cell lines, and in four biologic replicates for ascites.

### CD40L expression by flow cytometry

To assess cell surface expression of CD40L, 1 × 10^5^ cells were plated in triplicate in 12 well plates and maintained overnight. Cells were treated with the cell line specific IC_50_ MOI at 48 hours. The cells were incubated with MEM-288, Adv-GFP, or PBS. After 24 hours, media was aspirated, and cells were washed with PBS. After 24 hours, cells were trypsinized (Tryp-LE Express, Gibco 12604-013), counted and stained. Single stains and controls included CD40L and live/dead, GFP, and unstained. Cells were stained using LIVE/DEAD Fixable Violet Dead Cell Stain (Invitrogen, L34963, 1:200 in PBS), FC block (Anti-Mo CD16/32 [Invitrogen 14-0161-85] and purified anti-mouse CD16.2 [Biolegend, 149502], 1:100 in Flow Cytometry Staining Buffer [Invitrogen 00-4222-26]), anti-Mo CD154 (CD40 Ligand) (Invitrogen 12-1541-82, 1:40 in Flow Cytometry Staining Buffer]. Cells were fixed in Fixation Buffer (BD Biosciences, 554655) before analysis using a BD FACSCanto II flow cytometer. Data were analyzed using FlowJo software (TreeStar, Inc). Experiments were performed in biologic and triplicates for cell lines, and in four biologic replicates for ascites.

### IFNγ release assay

To assess IFNγ secretion by T-cells after viral treatment, 2 × 10^5^ human ascites cells were plated in triplicate each well of a round bottom 96 well plate and maintained overnight. The IC_50_ MOI at 48 hours (74) was selected as a treatment dose. MEM-288, Adv-GFP, or PBS was added to culture media. IFNγ levels in the supernatant were assessed at 48 hours with a human IFNγ specific ELISA (Mabtech ELISA Pro: Human IFN-γ, #34201HP-2).

### In vivo studies

Female FVB/N mice were purchased from Charles River Co. All animal experiments were approved by the Institutional Animal Care and Use Committee (IACUC, A175-19-08).

### In vivo imaging

Anesthetized animals (2 % isoflurane inhalation) underwent intraperitoneal injection of d-luciferin (150 mg kg^-1^; Regis Technologies, 1-360-243-200). Fluorescence was measured 5-10 minutes after luciferin injection with the IVIS Lumina XR camera (PerkinElmer).

### Endpoints

STOSE-luc cells were injected intraperitoneally (1 × 10^6^ cells in 200 μl) of female FVB/N mice aged 7-9 weeks. Rumors were confirming by *in vivo* imaging and mice were randomized to receive 1 × 10^9^ PFU in 100 μl intraperitoneal injections of either MEM288, Adv-GFP, or saline on days 12 and 15 after STOSE-luc cell injection. Mice were monitored with weights and *in vivo* imaging until day 27 when all mice were euthanized, and necropsies performed for immune endpoints.

For survival endpoints, treatment timing was selected when mice began to demonstrate signs of disease (determined by presence of ascites on clinical assessment). MEM288 or Adv-GFP given on days 15, 18, 46, and 49 after STOSE-luc cell injection. IG10-luc experiments were performed similarly, with 2 × 10^6^ cells in 200 μl injected into of female C57BL/6 mice aged 7 weeks and 1 × 10^9^ PFU in 100 μl intraperitoneal injections of either MEM288 or Adv-GFP given on days 46 and 49 after IG10-luc cell injection. Mice were subsequently monitored with weights and clinical assessments until death or predetermined humane endpoints, when mice were euthanized, and necropsies performed.

### Tissue processing

#### Tumor

All visible tumor was dissected off surrounding structures and weighed. A portion of tumor was placed in formalin. Remaining tumor was chopped into pieces. Collagenase A (Millipore Sigma COLLA-RO, 1 mg/mL) and DNAase1 (Sigma-Aldrich D5025, 10.8mg/mL) were added and the solution was placed in the shaker, filtered through a 40um filter and spun down. Red blood cell lysis with ACK lysis buffer (Gibco A10492) was performed 1-2 times. Cells were resuspended in PBS, counted, and normalized to a concentration of 1 × 10^7^/mL.

#### Spleen

Spleens were removed from euthanized mice and placed in complete media (RPMI 1640 with 10 % FBS, 2mM L-glutamine, 100U/ml penicillin and 100 ug/ml streptomycin) They were placed on top of a 100um strainer (Greinier Bio-One, #542000) and mashed with a rubber piston. Cells were spun down. Red blood cell lysis with ACK lysis buffer was performed 1-2 times per sample. Samples were spun down again and filtered through a 70um strainer (Greiner Bio-One, #542070).

#### IFNγ Enzyme-linked immunospot (ELISPOT) assay

Precoated 96-well plates (Mabtech Mouse IFN-γ ELIpot^PLUS^ kit, 3321-4HPT-2) were washed with PBS then blocked with complete media. Harvested effectors (splenocytes) were added to the plates in quadruplicate at 1 × 10^5^ cells/well with 1 × 10^5^ irradiated (100 Gy) STOSE-luc target cells (2 wells), positive control (1 well, phorbol myristate acetate (PMA) 1 ng/ml and ionomycin 1μM), negative control (1 well, additional complete medium). Additional control wells of irradiated STOSE-luc target cells alone and media alone were plated in duplicate. Plates were incubated for 40 hours. A biotinylated detection monocloPal antibody R4-6A2 at was added to each well (100ul/well, 1ug/mL in PBS with 0.5 % FBS), and incubated for 2 hours at room temperature. A dilute streptavidin-HRP (1:1000) was added to each well (100ul/well, 1:1000 in PBS-0.5 % FBS) and incubated for 1 hour. Vector NovaRED Substrate (100 ul/well Vector Laboratories, SK-4800) was added to each well and developed for 20 minutes. Color development was stopped by washing with deionized water. The plate was left to dry in the dark overnight. Plates were scanned and counted using the ImmunoSpot 7.0 Pro (Cellular Technology, Ltd., Cleveland, OH) The precursor frequency of tumor-specific T cells was determined by subtracting the background spots in target cells alone and effectors alone from the number of spots seen in response to tumor cells.

#### Flow cytometry for intratumoral immune cell characterization

Single cell suspensions of tumor cells were generated as described above. Single stains, live/dead, and unstained controls were used. Samples were plated in a 96 well round-bottom plate (1 × 10^6^ cells in 50ul, or entire sample), spun down, then incubated with live/dead cell stain, spun down and then incubated with FC block in Flow Cytometry Staining Buffer. Cells were then stained with an extracellular antibody cocktail in Brilliant Stain Buffer (ThermoFisher #566349) (Supplemental Table 1). For intracellular staining cells were fixed and permeabilized using 50µl of Fix Perm solution from eBioscience Foxp3 Transcription Factor Staining Buffer Set (ThermoFisher Scientific #00-5523-00), then spun down and resuspended in flow buffer.

Multicolor flow cytometry was performed in BD Fortessa 16 color analyzer. Data were analyzed using FlowJo software (TreeStar, Inc). Gating strategies for myeloid and lymphoid cell sets were adapted from published protocols (Supplemental Figure 2) [42].

#### Immunohistochemistry

Formalin-fixed, paraffin embedded tissues were evaluated via immunohistochemistry (IHC) for expression of CD3 (Dako, A0452 Rabbit mAb,) CD8 (Cell Signaling,98941T), CD31(Cell Signaling,77699) and Ki67(ABCam Technologies, AB1667). After a 1 hour, a TBS wash (Corning 46-012-CM) wash was performed. The sections were incubated in the secondary antibody ImmPRESS horse anti-rabbit IgG peroxidase polymer (VectorLabs, MP7401) stock concentrations were used and incubated for 30 minutes. The chromagen/substrate 3.3′- diaminobenzidine (ImmPACT DAB EqV substrate kit, Vector Laboratories, SK-4103) a DAB reaction was applied to visualize the location and intensity of the proteins of interest. The sections then were counterstained with a modified Lillie-Mayer hematoxylin (Biocare Medical, CATHE-M) and mounted.

A single pathologist (K.S.) was blinded to treatment groups performed analysis of immunohistochemistry slides. Analysis of CD3 and CD8 was performed by counting the number of CD3 or CD8 positive lymphocytes per high power field in three separate fields. CD31 was quantified as the number of vessels identified (defined as CD31 positive) per high power field in three separate fields. Ki-67 was quantified by manual count of the number of positive and negative cells for Ki67 within a high-power field in three separate fields, and then reported as a percentage of positive cells.

### Statistical analysis

All statistical analysis was undertaken in GraphPad Prism (v 9.3.1). Outliers were identified via the ROUT method. Data were tested for normality of distribution as determined by Anderson-Darling, D'Agostino & Pearson, Shapiro-Wilk, and Kolmogorov-Smirnov tests (data defined as non-normally distributed if determined to be non-normal by ≥ 2 tests). For comparisons with normally distributed data, statistical significance was determined by one-way ANOVA followed by Tukey's multiple comparison test (for ratio of largest to smallest standard deviation <2), or Brown Forsythe and Welch ANOVA with Dunnett's T3 multiple comparison test (for ratio of largest to smallest standard deviation ≥2). For non-normally distributed data, a Kruskal-Wallis test with Dunn's multiple comparison test was used for comparison. For comparison between two groups an unpaired t-test was used (with Welch's correction for non-normally distributed data). Survival curves were estimated using the Kaplan-Meier method and compared with the log rank (Mantel-Cox) test. Statistical significance was defined as *p* ≤ 0.05 (*), *p* ≤ 0.01 (**), *p* ≤ 0.001 (***), *p* ≤ 0.0001 (***).

## Results

### *In vitro* confirmation of MEM-288 infection of ovarian cancer cell lines

As a prelude to *in vivo* anti-tumor studies, we confirmed MEM-288 was able to infect human epithelial ovarian cancer cell lines *in vitro*. Infection was confirmed by assessing for expression of the MEM-288 transgenes demonstrated by IFNβ in the supernatant of infected cells and MEM40 (CD40L) on the cell surface.

MEM-288 treatment was compared to control treatments of phosphate-buffered saline (saline) or green-fluorescent labelled adenovirus (Adv-GFP). Adv-GFP is an oncolytic adenovirus but did not contain the transgenes CD40L or IFNβ. These controls were selected to help differentiate between the effect specific to experimental MEM-288 treatment, versus generalized class effect of oncolytic viruses, versus placebo.

**Fig. 1:**
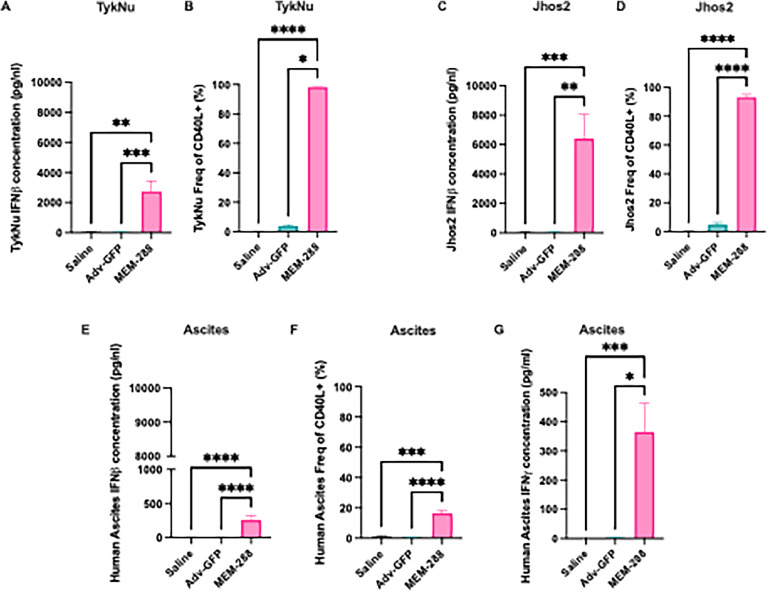
MEM-288 infection causes transgene expression in human ovarian cancer cell lines and human ascites samples. (A-B) TykNu cells demonstrated elevated concentrations of (A) IFNβ (*p* = 0.00001) and (B) expression of CD40L (*p* = 0.0001) after exposure to MEM-288, compared to Adv-GFP and saline exposure. (C-D) Jhos2 cells demonstrated elevated concentrations of (A) IFNβ (*p* = 0.00001) and (B) expression of CD40L (*p* = 0.0001) after exposure to MEM-288, compared to Adv-GFP and saline exposure. (E-F) Primary human ascites samples demonstrated elevated concentrations of (E) IFNβ (*p* < 0.0001) and (F) Expression of CD40L (*p* < 0.0001) after exposure to MEM-288, compared to Adv-GFP and saline exposure. (G) Human ascites samples containing both tumor and immune cells demonstrated increased concentrations of IFNγ, a marker of T-cell activation when exposed to MEM-288, compared to Adv-GFP or saline (*p* = 0.0002). All error bars represent standard error of the mean (SEM). All experiments were performed in triplicate or quadruplicate. Statistical significance was defined as *p* ≤ 0.05 (*), *p* ≤ 0.01 (**), *p* ≤ 0.001 (***), *p* ≤ 0.0001 (***).

MEM-288 infection led to IFNβ secretion in the culture supernatant in the TykNu and Jhos2 cell line. In TykNu cells, MEM-288-treated cells had higher mean concentrations IFNβ (MEM-288 941 pg/nl, Adv GFP 52 pg/nl, saline 58 pg/nl, *p* = 0.0001) (Fig. 1A). Jhos2 cells demonstrated secretion of IFNβ into the supernatant at a mean concentration of 6400 pg/nl, versus 35 pg/nl for Adv-GFP and 33 pg/nl for saline (*p* = 0.0001) (Fig. 1C).

MEM-288 infection significantly increased CD40L expression in human cell lines TykNu and Jhos2. Specifically, MEM-288 infection resulted in 98 % of TykNu cells expressing CD40L, significantly more than controls (Adv-GFP 4 %, saline 0 %, *p* = 0.0001) (Fig. 1B). Similar expression levels of CD40L were quantified in Jhos2 cell line; control treatment had minimal induction of CD40L (Adv-GFP 5 %, saline 0 %) whereas MEM-288 treatment had significantly higher CD40L expression (MEM-288 93 % *p* = 0.0001) (Fig. 1D).

Viral-mediated oncolysis following MEM-288 infection was also demonstrated in both human and mouse ovarian cancer cell lines. The IC_50_ MOI, defined as the dose (multiplicity of infection) of virus causing death in 50 % of cells at 48 hours, for each cell line was determined by cell viability assay (Supplemental Fig. 1). In multiple cell lines, MEM-288 demonstrated enhanced cell lysis compared to control Adv-GFP, a previously described feature of MEM-288.

### MEM-288 immune activation in primary human ascites

We next determined whether MEM-288 could infect and generate an immune response in primary human ascites samples *in vitro*. Ascites represents a unique tumor microenvironment that contains a heterogeneous mixture of tumor cells of different clonotypes, as well as immune cells and cytokines. As such, cryopreserved human ascites samples obtained from patients with epithelial ovarian cancer represent a more biologically diverse and accurate *in vitro* model.

MEM-288 infection of thawed and suspension-cultured ascites resulted in increased secretion of IFNβ (mean concentration of 257 pg/nl in MEM-288, 0 pg/nl in Adv-GFP, 0 pg/nl in saline-treated controls, *p* < 0.0001) (Fig. 1E) and increased CD40L expression (16 % for MEM-288, versus 1 % in Adv-GFP, 1 % saline-treated controls, *p* < 0.0001) (Fig. 1F).

The ability of MEM-288 to induce effector lymphocyte activation (e.g. T-cells and/or NK cells) was assessed by IFNγ ELISA. Ascites cells were exposed to MEM-288, Adv-GFP, or saline as described above. At 48 hours after initial infection, culture media was evaluated for IFNγ levels as a marker effector cell activation. Human ascites samples exposed to MEM-288 had significantly higher levels of IFNγ versus controls (median concentration 365 pg/nl for MEM-288, 3 pg/nl for Adv-GFP, 0 pg/nl for saline, *p* = 0.0002) (Fig. 1G).

### Intraperitoneal MEM-288 generates an *in vivo* systemic tumor-specific immune response and potent ascites volume reduction

The evidence than MEM-288 promotes an *in vitro* T-cell activation within ascites naturally led us to test the immune activation and anti-tumor capacity of MEM-288 in an immune competent ovarian cancer mouse model.

Luciferase-labelled mouse ovarian cancer cells (STOSE-luc) were injected intraperitoneally in immune competent FVB/N mice. When intraperitoneal tumors were confirmed by *in vivo* imaging, mice were randomized into treatment groups of either MEM-288, Adv-GFP, or saline administered intraperitoneally on days 12 and 15 after STOSE-luc cell injection. This time frame was selected based on previously published data in mouse models of lung cancer [29], to align treatment with mild to moderate tumor burden, but before the onset of clinical deterioration and end-organ dysfunction. We euthanized the mice two weeks after treatment (day 27 after STOSE-luc cell injection) to allow time to mount an anti-tumor immune response. Ascites, solid tumors, and spleens were processed.

**Fig. 2:**
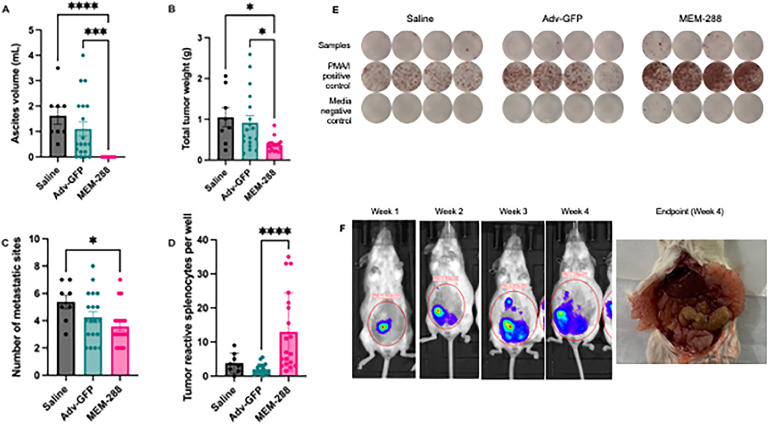
**Anti-tumor and immune stimulatory activity of MEM-288 *in vivo*.** (A-C) Two weeks after intraperitoneal treatment with saline, control oncolytic virus (Adv-GFP), or MEM-288, mice were assessed for tumor burden as measured by (A) ascites volume, (B) tumor weight, and (C) number of metastatic sites. (D) Generation of a systemic anti-tumor immune response was evaluated by IFNγ ELISA, measuring number of tumor-reactive splenocytes in mice treated with saline, Adv-GFP, or MEM-288. (E) Representative wells from IFNγ ELISPOT with each column representing a biologic replicate. Splenocytes were placed in duplicate (not shown) with irradiated STOSE-luc target cells in sample wells. Positive control wells contained splenocytes with a non-specific T-cell stimulator (PMA/I, phorbol myristate acetate/ ionomycin). Negative control wells contained splenocytes with media. Number of spots per well was quantified, with each spot representing a tumor-reactive T-cell. (F) Representative images are shown from imaging experiments. Mice were monitored with *in vivo* imaging at least weekly from time of cell injection (day 0) to experimental endpoint (day 27). The experimental endpoint was selected to be 2 weeks after treatments were administered as the control mice (saline, Adv-GFP) approached humane endpoints, with large abdominal ascites and tumor burden on necropsy. Error bars represent standard error of the mean (SEM) with each point representing a biologic replicate. Statistical significance was defined as *p* ≤ 0.05 (*), *p* ≤ 0.01 (**), *p* ≤ 0.001 (***), *p* ≤ 0.0001 (***).

MEM-288 treatment was associated with a significant reduction in ascites volume (mean ± [standard deviation] SD; 0.0 mL ± 0.0 mL) compared to Adv-GFP (1.1 mL ± 1.2 mL) and saline (1.6 mL ± 0.9 mL) (*p* < 0.0001). Solid tumor weight was decreased in mice treated with MEM-288 (0.37 g ± 0.17 g) compared to Adv-GFP (0.91g ± 0.72 g) and saline (1.1g ± 0.66 g) (*p* = 0.004). When specifically comparing MEM-288 to Adv-GFP correcting for multiple comparisons, there remained a statistically significant decrease in ascites volume (*p* = 0.0002) and tumor weight (*p* = 0.01) with MEM-288 treatment. The number of metastatic sites was similar between all treatment groups with MEM-288 treated mice having the fewest metastatic sites (defined as number of organs affected; 3.5 ± 1.4), followed by Adv-GFP (4.2 ±1.7), and saline (5.3 ±1.4) (*p* = 0.03). There was no different in number of metastatic sites when MEM-288 and Adv-GFP were directly compared (*p* = 0.66) (Fig. 2A-C).

Mouse splenocytes were utilized to assess for a systemic anti-tumor immune response using an IFNγ enzyme-linked immunospot (ELISPOT) assay. Splenocytes (effector cells) were thawed and co-cultured with irradiated STOSE-luc (target cells) and an IFNγ ELISA technique was used to assess T-cell activation in response to exposure to tumor cells [30]. Positive control wells contained splenocytes with a non-specific T-cell stimulator (PMA/I, phorbol myristate acetate/ ionomycin). Negative control wells contained splenocytes with media. Each spot within a well represents a splenocyte secreting IFNγ in response to target cells, suggesting the presence of systemic tumor-reactive T-cells (Fig. 2E). MEM-288 treated mice had an increased number of spots (representing tumor-reactive splenocytes) (13 ± 11) compared to Adv-GFP (2 ± 1.5) and saline (4 ± 3) (p < 0.0001). Specifically, there was a significant increase in tumor-reactive splenocytes for MEM-288 treated mice versus Adv-GFP that remained statistically significant on test of multiple comparisons (p <0.0001) (Fig. 2D).

### MEM-288 tumor microenvironment (TME) modulation increases M1:M2 macrophage phenotypes and decreased angiogenesis

We sought to explore the mechanism of anti-tumor activity by evaluating the tumor microenvironment. Mice were monitored with weights and *in vivo* imaging until day 27 when all mice were euthanized tumors were dissected and processed into single cell suspensions.

**Fig. 3:**
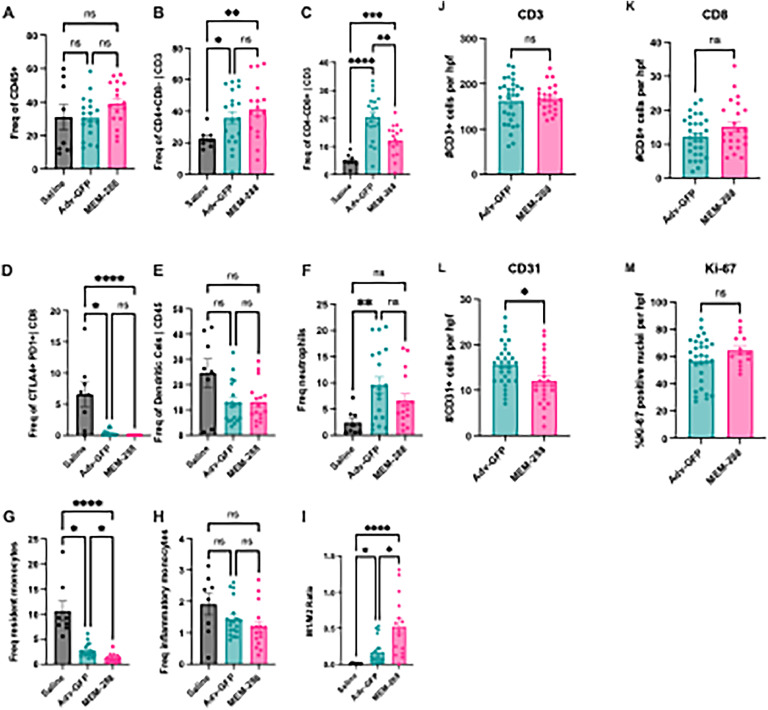
**MEM-288 and Adv-GFP altered the tumor microenvironment *in vivo.*** (A-I) Proportions of cell populations were assessed with flow cytometry. There was no significant difference in (A) immune cells (CD45+) between treatment groups. There was a significant increase in proportion of (B) CD4+ T-cells with MEM-288 vs saline (*p* = 0.004) and Adv-GFP vs saline (*p* = 0.02). (C) CD8+ T-cells were increased in Adv-GFP compared to saline (*p* < 0.0001) and MEM-288 (*p* = 0.003). Proportions of (D) CTLA4+ and PD1+ CD8 T-cells were decreased with MEM-288 versus saline (*p* < 0.0001) and Adv-GFP versus saline (*p* = 0.02) but not significantly different between Adv-GFP and MEM-288. MEM-288 treatment did not affect proportions of (E) dendritic cells or (F) neutrophils compared to saline. Proportions of (G) resident monocytes were lower with MEM-288 treatment compared to Adv-GFP (*p* = 0.01) and saline (*p* < 0.0001) with no difference in (H) inflammatory monocytes. The (I) ratio of M1 “anti-tumor” macrophage phenotype: M2 “pro-tumor” macrophage phenotype was increased with MEM-288 treatment versus Adv-GFP (*p* = 0.05) and saline (*p* < 0.0001). (J-M) Immunohistochemistry (IHC) was used to quantify (J) CD3+ and (K) CD8+ cells as an average number of positive staining cells per high power field. There was no difference in CD3 or CD8 staining between treatment groups. (L) CD31 staining, defined as the number of vessels per high power field staining positive for CD31, was decreased with MEM-288 treatment versus Adv-GFP (*p* = 0.02). (M) Ki-67 index was not different between treatment groups. Error bars represent standard error of the mean (SEM) with each point representing a biologic replicate. Statistical significance was defined as *p* ≤ 0.05 (*), *p* ≤ 0.01 (**), *p* ≤ 0.001 (***), *p* ≤ 0.0001 (***).

Immune populations within the tumor microenvironment were characterized using flow cytometry (Supplemental Table 1). There were no significant differences in proportion of immune cells (CD45+) between the groups (Fig. 3A). The proportion of CD4+ T-cells (Fig. 3B) and CD8+ T-cells (Fig. 3C) were increased after treatment with Adv-GFP and MEM-288 compared to saline. CD8+ T-cells were decreased with MEM-288 treatment compared to Adv-GFP. Expression of CTLA4 and PD1 on CD8+ T-cells was significantly decreased with Adv-GFP and MEM-288 treatment, but there was no significant difference in proportion of CTLA4+/PD1+ cells between Adv-GFP and MEM-288 treated tumors (Fig. 3D). There were no differences in the proportion of dendritic cells (Fig. 3E) or neutrophils (Fig. 3F) between MEM-288 and control treatment. There were fewer resident monocytes present with MEM-288 treatment, compared to Adv-GFP and saline (*p* < 0.0001) (Fig. 3G). There was no difference in inflammatory monocytes (Fig. 3H). MEM-288 treated mice had a higher ratio M1:M2 macrophage phenotypes (“anti-tumor”: “pro-tumor”) (Fig. 3I).

There was no significant difference in CD3+ (Fig. 3J) or CD8+ (Fig. 3K) cells on immunohistochemistry (IHC) between MEM-288 and Adv-GFP treated tumors. There was a significant decrease in the number of angiogenic vessels identified by CD31 expression per high power field in MEM-288-treated tumors compared to Adv-GFP (*p* = 0.02) (Fig. 3L). There was no difference in Ki-67 index between MEM-288 and Adv-GFP treated tumors (Fig. 3M).

**Fig. 4:**
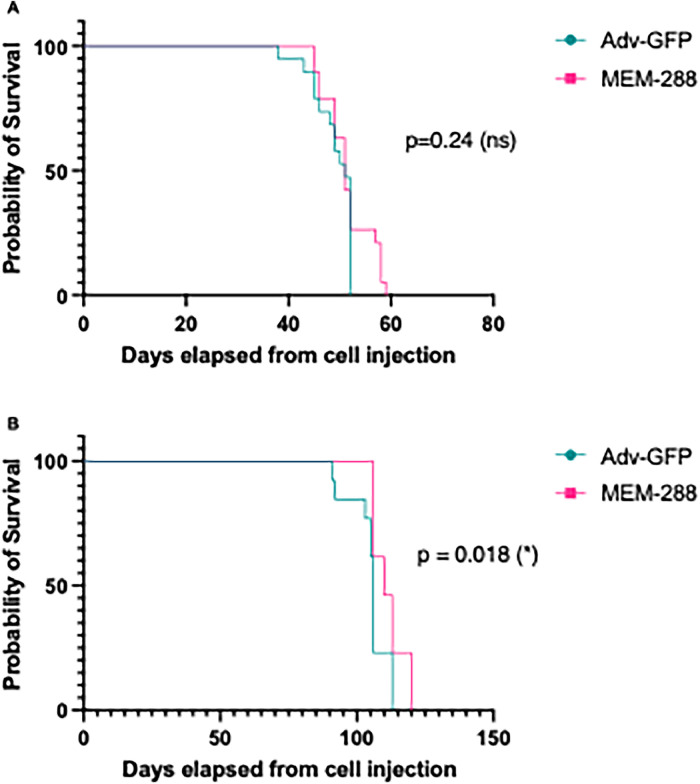
Efficacy of MEM-288 *in vivo* in epithelial ovarian cancer mouse models (A) STOSE-luc cells (1 × 10^6^) were injected intraperitoneal (i.p.) and tumor formation confirmed via *in vivo* imaging on day 10. Mice were randomized to MEM-288 or Adv-GFP i.p. treatment was administered on days 15, 18, 46, and 49. Median survival was 51 days for Adv-GFP and 51 days for MEM-288 (*p* = 0.24). (B) IG10-luc cells (2 × 10^6^) were injected intraperitoneal (i.p.) and tumor formation confirmed via *in vivo* imaging. Mice were randomized to MEM-288 or Adv-GFP i.p. treatment was administered on days 46 and 49. Median survival was 106 days for Adv-GFP and 110 days for MEM-288 (*p* = 0.018). Significance was assessed using the log-rank (Mantel-Cox) tests. Statistical significance was defined as *p* ≤ 0.05 (*), *p* ≤ 0.01 (**), *p* ≤ 0.001 (***), *p* ≤ 0.0001 (***).

Intraperitoneal treatment with single agent MEM-288 was tested for survival benefit in two immune competent mouse models. In the first model, STOSE-luc cells were injected intraperitoneally into FVB/N mice and tumor formation confirmed by *in vivo* imaging on day 10. Mice received MEM-288 (*n* = 19) or Adv-GFP (*n* = 19) intraperitoneally at early stage of disease (days 15, 18 post STOSE-luc injection) and late-stage disease (days 46, 49). Median survival was 51 days (range 45-58) for MEM-288 mice and 51 days (range 38-52) in the Adv-GFP mice (*p* = 0.24) (Fig. 4A). In the second model, IG10-luc cells were injected intraperitoneally into C57BL/6 mice. The natural history of IG10-luc tumor growth is slower [31]. The mice were treated with either intraperitoneal MEM-288 (*n* = 13) or Adv-GFP (*n* = 14) after development of early clinical signs of carcinomatosis (days 46 and 49). Median survival was 106 days (range 91-113) for Adv-GFP mice and 110 days (range 106-120) for MEM-288 mice (*p* = 0.018) (Fig. 4B).

## Discussion

Innovative therapeutic strategies for patients with advanced stage and recurrent epithelial ovarian cancer are urgently needed, as this disease is not inherently responsive to immunotherapy and all patients with recurrent disease eventually develop resistance to chemotherapy. Due to the intraperitoneal pattern of spread, administration of intraperitoneal therapy has been a promising modality for drug delivery, however toxicity of intraperitoneal chemotherapies remains a major barrier to widespread adoption [[2], [3], [4]].

Oncolytic viruses are typically administered via direct intratumoral injections due to concern for rapid eradication by the immune system with intravenous administration. For therapies such as Talimogene Laherparepvec (T-VEC) used in advanced melanoma, metastatic lesions and relatively easy to administer as cutaneous lesions are often present [32]. In other diseases where tumors are not as easily accessible, intratumoral administration is more cumbersome, requiring a separate surgical procedure. Intraperitoneal administration of oncolytic viruses for treatment of malignancies with primarily intraabdominal spread is a promising strategy currently being tested with T-VEC [33].

MEM-288 demonstrated anti-tumor activity and the ability to generate a systemic anti-tumor immune response after intraperitoneal administration. While both solid tumor burden and ascites burden were significantly decreased with MEM-288 treatment compared to Adv-GFP or placebo, the effect on ascites reduction was dramatic. Ascites reduction is a clinically relevant outcome. The accumulation of large volume ascites in patients with ovarian cancer requires frequent procedures for drainage and is a detriment to quality of life and nutritional status. The mechanism of ascites accumulation is multifactorial, but is thought to be driven primarily by increased vascular permeability with or without lymphatic obstruction [34]. For this reason, bevacizumab, a monoclonal antibody targeting vascular endothelial growth factor A (VEG-F) is effective for ascites reduction [35], but this drug also has a significant toxicity profile including severe hypertension and bowel perforation that often precludes its use. MEM-288 may have significant clinical value in its ability to reduce ascites. Combination therapy with oncolytic viruses is an important area of future study that may improve clinical efficacy and overall survival, as immunotherapy is often synergistic with chemotherapy in other gynecologic malignancies [11,12,36].

We demonstrated that MEM-288 can generate a systemic anti-tumor immune response using an ELISPOT assay. While this abscopal effect has been demonstrated with intra-tumoral injection of oncolytic viruses [37], we demonstrated systemic immune response can also be generated with intraperitoneal administration.

Many questions remain regarding the mechanism of action of MEM-288 in ovarian cancer. Analysis of immune cell populations within ascites and solid tumors was planned, however because of the dramatic reduction in ascites with MEM-288 treatment, analysis of ascites was not feasible. We hypothesize that enrichment of CD8+ T-cells and dendritic cells within malignant ascites may play a role. A small study in patients with malignant ascites and gastrointestinal cancers also utilized a type 5 oncolytic adenovirus with intraperitoneal administration. This study demonstrated 75 % ascites control rate with no grade 3-4 adverse effects. Mass cytometry of ascites after treatment suggested tumor cell depletion and increased dendritic cell and CD8+ T-cells within ascites [38].

While we expected to see an increase in CD8+ T-cells within solid tumors, we did not see a higher proportion of these cells in MEM-288 treated mice compared to controls. Oncolytic viral therapy (MEM-288 and Adv-GFP) appeared to decrease T-cell inhibitory markers such as CTLA4 and PD1. However, because this was seen with both the MEM-288 and Adv-GFP compared to saline, we suspect this is due to a class effect of oncolytic viruses rather than a direct effect of transgene expression in cells infected with MEM-288. The tumor microenvironment contained a higher proportion of macrophages with an anti-tumor phenotype, suggesting that innate immune populations may play a role in the mechanism of MEM-288. CD40L can activate macrophages and induce an anti-tumor innate immune response in addition to activation of CD8+ T-cells [39]. Ultimately, the mechanism of this therapy may be different either due a unique aspect of the biology of ovarian cancer or the modality of administration. Further studies to elucidate mechanism are warranted and may include repeat *in vivo* studies after depletion of CD8+ T-cells to determine if the effect is T-cell dependent.

While much of this work is within a mouse model of epithelial ovarian cancer, we also demonstrated that MEM-288 reliably infects human ovarian cancer cell lines and primary samples obtained from patient ascites, with expression of both CD40L and IFN beta transgenes. In addition, *in vitro* work with human ascites that contains both tumor cells and immune cells, showed that MEM-288 was able to generate a T-cell response *in vitro* in primary human ascites samples.

Together, these data suggest that MEM-288 is a promising novel therapeutic in epithelial ovarian cancer that warrants further preclinical investigation to understand mechanism of action. This agent has already been demonstrated to be safe in humans in phase I clinical trials. This work suggests that patients with epithelial ovarian cancer may also benefit from this agent. Pharmacokinetic studies of the intraperitoneal route of administration are worthy of investigation due to the relative ease of administration compared to direct intratumoral injection, and possibility of increased efficacy in ovarian cancer due to the pattern of disease spread.

## CRediT authorship contribution statement

**Pamela N. Peters:** Conceptualization, Data curation, Formal analysis, Funding acquisition, Investigation, Methodology, Project administration, Resources, Software, Validation, Validation, Writing – original draft, Writing – review & editing. **Regina S. Whitaker:** Data curation, Methodology, Project administration, Writing – original draft, Writing – review & editing, Conceptualization. **Felicia Lim:** Formal analysis, Resources, Software, Writing – review & editing. **Shonagh Russell:** Formal analysis, Methodology, Resources, Supervision, Writing – review & editing, Visualization. **Elizabeth A. Bloom:** Data curation, Writing – review & editing. **Justin Pollara:** Methodology, Resources, Software, Writing – review & editing. **Kyle C. Strickland:** Formal analysis, Resources, Writing – review & editing. **Mark J. Cantwell:** Resources, Writing – review & editing, Supervision. **Amer Beg:** Methodology, Resources, Writing – review & editing, Conceptualization, Supervision. **Andrew Berchuck:** Funding acquisition, Resources, Writing – review & editing. **Scott Antonia:** Conceptualization, Funding acquisition, Resources, Supervision, Writing – review & editing. **Rebecca A. Previs:** Conceptualization, Data curation, Funding acquisition, Investigation, Methodology, Project administration, Resources, Supervision, Writing – original draft, Writing – review & editing.

## Declaration of competing interest

The authors declare the following financial interests/personal relationships which may be considered as potential competing interests:

Mark Cantwell is an employee, stock owner of Memgen, Inc and has a patent on MEM-288.

## Appendix Supplementary materials

Image, application 1
