# Supplementary material for: Oncolytic adenovirus MEM-288 encoding membrane-stable CD40L and IFNβ induces an anti-tumor immune response in high grade serous ovarian cancer
Source: Neoplasia. 2024 Sep 13;57:101056. doi: 10.1016/j.neo.2024.101056 (PMC11417341; doi:10.1016/j.neo.2024.101056)
Supplement: Supplementary file 1 [file mmc1.docx]

**Supplemental Figures and Tables**

**Supplemental Figure 1.** **Cell viability assays for human and mouse ovarian cancer cell lines 48 hours after treatment with MEM-288 or Adv-GFP.** All assays performed with technical triplicates and repeated in three biologic triplicates, with one representative replicate shown for each cell line. IC_50_ doses of MEM288 were determined by logarithmic transformation of multiplicity of infection (MOI) followed by non-linear regression (variable slope, four parameter): **A)** TykNu: 39. **B)** Jhos2: 173. **C)** STOSE-luc: 89. **D)** IG10luc: 64.

| **Marker** | **Channel** | **Company** | **Catalogue no** |
| --- | --- | --- | --- |
|  |  |  |  |
| **Dendritic Cell /Myeloid panel** | |  |  |
| CD19 | Alexa Fluor 488 (FITC) | Biolegend | 115521 |
| CD64 | PerCP-Cy5.5 | Biolegend | 139308 |
| F4/80 | APC | Biolegend | 123116 |
| CD206 | APC-Alexa 700 | Biolegend | 141734 |
| CD11c | APC-Cy7 | BD Biosciences | 561241 |
| CD24 | BUV496 | BD Biosciences | 612953 |
| CD86 | BV510 | Biolegend | 105039 |
| CD80 | BV650 | Biolegend | 104732 |
| Ly6C | BV711 | Biolegend | 128037 |
| Ly6G | BV786 | Biolegend | 127645 |
| CD11b | PE | Biolegend | 101208 |
| CD103 | PE-Texas Red | Biolegend | 121429 |
| B220 | PE-Cy5 | Biolegend | 103209 |
| MHC-II | PE-Cy7 | ThermoFisher Scientific | 25-5321-80 |
|  |  |  |  |
| **T-cell panel** |  |  |  |
| CD3 | PerCP-Cy5.5 | BD Biosciences | 560527 |
| gd TCR | APC | Biolegend | 118116 |
| CD44 | APC-Alexa 700 | Biolegend | 103026 |
| CD69 | APC-Cy7 | Biolegend | 104526 |
| TIM3/CD366 | BV421 | Biolegend | 134019 |
| PD1 | BV510 | Biolegend | 135241 |
| CD8a | BV650 | Biolegend | 100742 |
| IFNg | BV711 | Biolegend | 505836 |
| CD4 | BV786 | Biolegend | 100552 |
| FoxP3 | PE | ThermoFisher Scientific | 12-5773-80 |
| CTLA4/CD152 | PE-Texas Red | Biolegend | 106317 |
| CD62L | PE-Cy5 | Biolegend | 104410 |
| GzB | PE-Cy7 | Biolegend | 372214 |
|  |  |  |  |
| **Eosinophil/endothelial panel** | |  |  |
| VEGF/CD309 | PerCP-Cy5.5 | Biolegend | 121918 |
| Siglec-F | APC | Biolegend | S17007L |
| CD11b | APC-Alexa 700 | Biolegend | 101222 |
| CD31 | APC-Cy7 | Biolegend | 102534 |
| Ly6C | BV711 | Biolegend | 128037 |
| Ly6G | BV786 | Biolegend | 127645 |
| CD34 | PE/Dazzle594 | Biolegend | 119330 |
| MHC-II | PE-Cy7 | ThermoFisher Scientific | 25-5321-80 |
|  |  |  |  |
| **For all panels** |  |  |  |
| CD45 | BV605 | Biolegend | 103155 |
| Live/Dead | BV421 | ThermoFisher Scientific | L34963 |
| Live/Dead | BUV496 | ThermoFisher Scientific | L34961 |
| CD16/CD32 | Fc blocker | Invitrogen | 14-0161-82 |
| CD16.2 | Fc blocker | Biolegend | 149502 |
| Brilliant Stain Buffer |  | BD Biosciences | 566349 |

**Supplemental Table 1. Antibody panel for in flow cytometry analysis of STOSE-luc tumors after treatment with MEM-288, Adv-GFP, or saline.**

**
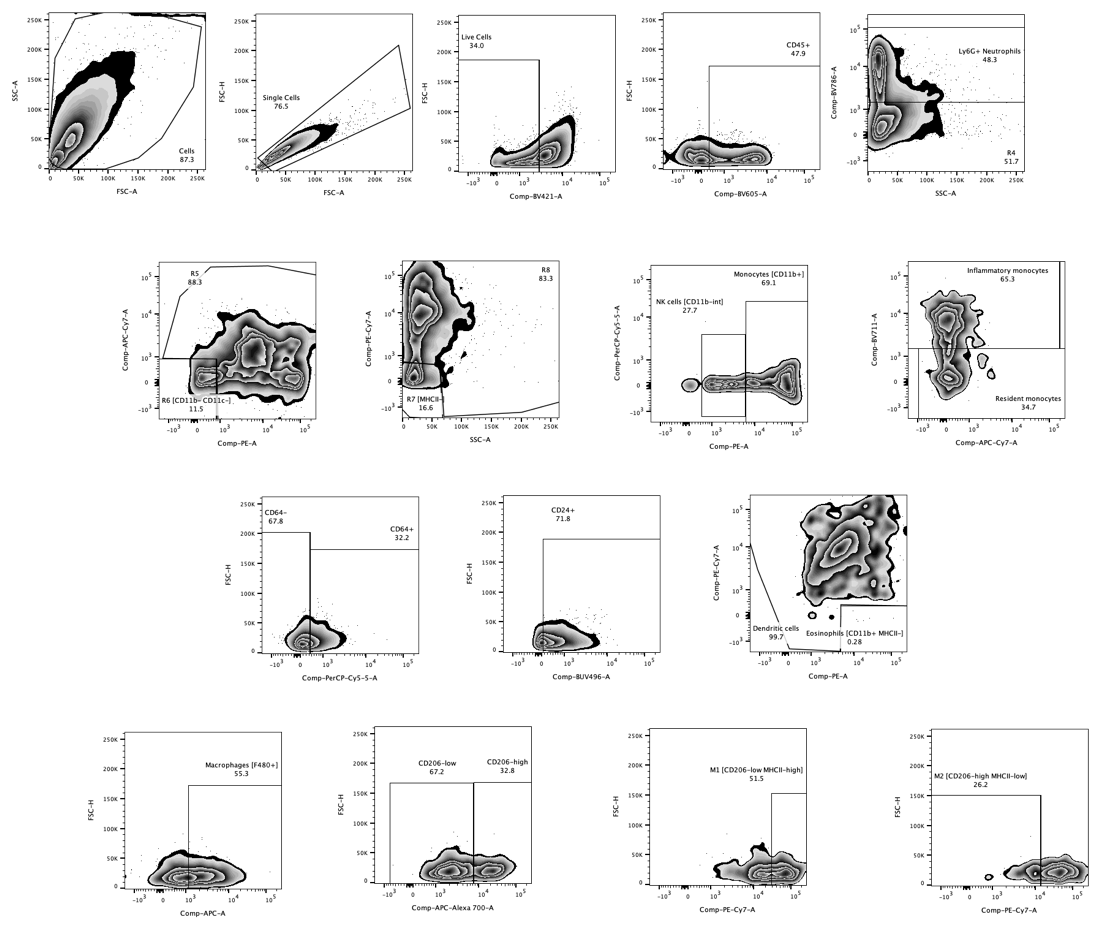
**

A

**
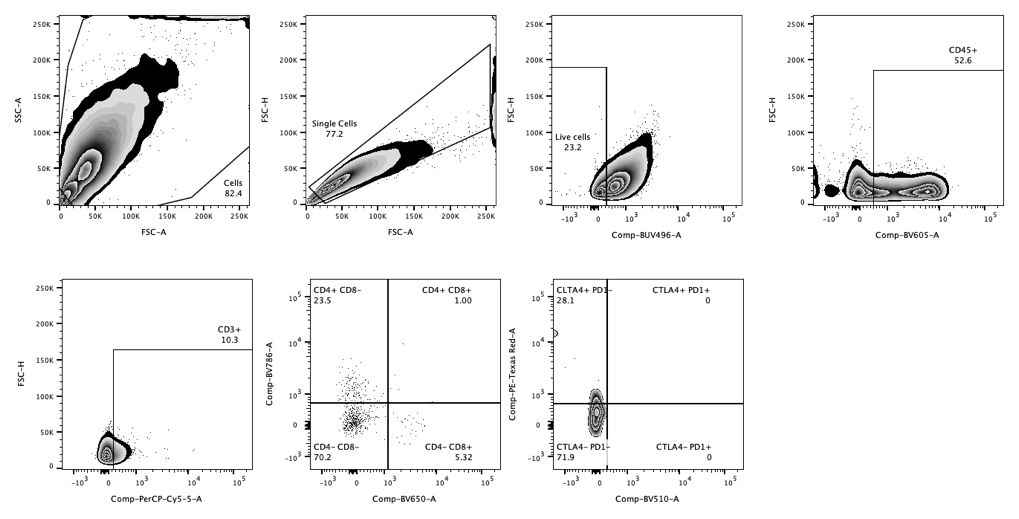
**

B

**Supplemental Figure 2. Flow cytometry gating strategies for myeloid and lymphoid cell subsets. A)** Myeloid cell panel - cells were isolated from enzymatically digested tumor cells, and after the exclusion of doublets, debris, and dead cells, live immune cells were identified by CD45 staining. A sequential gating strategy was then used to identify populations that express specific markers. Gates containing multiple cell populations are numbered (R1-R8). Gates containing a single cell population are labeled with the included cell type. These cell populations include neutrophils, NK cells, inflammatory and resident monocytes, dendritic cells, eosinophils, and macrophages (both M1 and M2). **B)** Lymphoid cell panel - cells were isolated from enzymatically digested tumor cells. Debris, doublets, and dead cells were excluded, and then live immune cells were identified by CD45 staining. A sequential gating strategy was then used to identify populations that express specific markers. These cell populations include CD8+ and CD4+ T-cells, and then they were further divided based on their CTLA4 and PD1 expression.

FSC, forward scatter; MHC-II, major histocompatibility complex class I; SSC, side scatter.
